# Combination of Cancer Stem Cell Markers CD44 and CD24 Is Superior to ALDH1 as a Prognostic Indicator in Breast Cancer Patients with Distant Metastases

**DOI:** 10.1371/journal.pone.0165253

**Published:** 2016-10-21

**Authors:** Yoshiya Horimoto, Atsushi Arakawa, Noriko Sasahara, Masahiko Tanabe, Sei Sai, Takanori Himuro, Mitsue Saito

**Affiliations:** 1 Department of Breast Oncology, Juntendo University School of Medicine, Tokyo, Japan; 2 Pathology and Oncology, Juntendo University School of Medicine, Tokyo, Japan; 3 Human Pathology, Juntendo University School of Medicine, Tokyo, Japan; 4 Department of Breast Surgery, Kyoundo Hospital, Tokyo, Japan; 5 Medical Physics Research Program, Research Center for Charged Particle Therapy, National Institute of Radiological Sciences, Chiba, Japan; Second University of Naples, ITALY

## Abstract

The combination of CD44 and CD24, or aldehyde dehydrogenase 1 (ALDH1) alone, is a widely used cancer stem cell marker in breast cancer. However, no conclusion has yet been reached as to which marker is the best for characterizing cancer stemness. Immunohistochemical evaluation using cancer stem cell markers is clearly less common clinically than in basic experiments and how the expressions of these markers relate to patient outcomes remains controversial. To investigate whether combining these markers might improve the prediction of patient outcomes, we immunohistochemically examined clinical samples. Primary invasive breast cancer samples from 61 patients who eventually developed distant metastases after curative surgery were immunohistochemically examined. All patients were free of metastatic disease at the time of surgery and received standard adjuvant systemic treatments. CD44^+^/24^-^ and ALDH1-positive rates in primary tumors differed according to intrinsic subtype. ER-positive patients with CD44^+^/24^-^ tumors had significantly longer disease-free-survival than all other ER-positive patients (p = 0.0047). On the other hand, CD44^+^/24^-^ tumors were associated with poor outcomes of ER-negative patients (p = 0.038). Finally, expression patterns of CD44 and ALDH1 in single tumors were strikingly different and there were virtually no individual double-stained cells. Thus, this combination does not allow evaluation of relationships with patient outcomes. Our results raise the possibility of CD44^+^/24^-^ being a good prognostic marker, one which would allow treatment effects and outcomes to be predicted in patients with recurrent breast cancer.

## Introduction

Cancer stem cells (CSCs) are defined by having the potentials to replicate and to form tumors [1–3]. Based on a number of basic studies, the combination of CD44 and CD24, or aldehyde dehydrogenase 1 (ALDH1) alone, is a widely used CSC marker in breast cancer [4, 5]. For instance, CD44-positive and CD24-negative (CD44^+^/24^-^) cells sorted according to these markers can form tumors after subcutaneous injection into immunodeficient mice[6]. Similarly, only CD44^+^/24^-^ cells from breast cancer cell lines were able to form lung metastases in the *in vivo* experiments of another study [7]. Moreover, disseminated tumor cells in the bone marrow of breast cancer patients were shown to be enriched with CD44^+^/24^-^ cells [8].

Notably, populations of CD44^+^/24^-^ and ALDH1-positive cells rarely correspond to each other. For instance, Liu et al. revealed that only 0.7% of CD44^+^/24^-^ cells were positive for ALDH1 in SUM149 and MCF7 cells [9]. Another study indicated that such double positive cells (CD44^+^/24^-^/ALDH1^+^) showed highly enhanced tumorigenicity and metastasis *in vitro* [10]. However, no conclusion has yet been reached as to which marker would be preferable for characterizing cancer stemness. Researchers generally choose one of these markers for study, such that totally different populations of cells might actually be examined according to CSC markers.

It is generally accepted that the more CSC markers a tumor expresses, the more aggressive the tumor tends to be, as reflected by resistance to systemic therapies and poor outcomes [11–13]. However, immunohistochemical (IHC) evaluation using CSC markers is clearly less common than *in vitro* and *in vivo* experiments [14–18]. We previously showed that ALDH1 expression was more common in early recurrence cases with ER-positive/human epidermal growth factor receptor (HER2)-negative breast cancer but how the expressions of these markers relate to patient outcomes is still controversial. Moreover, only a few studies have simultaneously measured protein expressions of CD44, CD24 and ALDH1 in clinical samples investigated employing IHC [14, 16, 17, 19].

We hypothesized that CD44^+^/24^-^ and ALDH1-positive cells, evaluated with IHC, differ within single tumors and that combining these markers might improve the prediction of patient outcomes.

## Materials and Methods

### Patient samples

Primary invasive breast cancer samples from 61 patients who eventually developed distant metastases, months to years after having undergone curative surgery, and who were then treated for recurrent diseases during the 2006 to 2013 period at Juntendo University Hospital, were retrospectively examined employing IHC. Clinicopathological features of these patients are shown in Table 1. All patients were free of metastatic disease at the time of surgery and received standard adjuvant systemic treatments, endocrine agent administration and/or chemotherapy. Subtypes of primary tumors were; luminal, defined by being estrogen receptor (ER) and/or progesterone receptor (PgR)-positive, in 62% (38 cases), HER2 in 10% (6) and triple negative (TN) in 28% (17). The median disease-free-survival (DFS) and overall survival (OS) were 30 and 60 months, respectively.

**Table 1:** Clinicopathological features of the 61 patients.

Characteristics		
Age (median)		55
		(29–75)
Histology (n)	IDC	57
	ILC	4
pStage (n)	I	4
	II	40
	III	17
Subtype (n)	Luminal*	38
	HER2	6
	TN	17
Systemic adjuvant therapy (n)		
	CT+ET	32
	CT alone	18
	ET alone	11
First metastatic sites (n)		
	Bone	16
	Liver	11
	Lungs	13
	Pleura	5
	GI	4
	LN	13
	Skin	4
	Others	4

n: number of patients

*includes five HER2-positive cases

IDC: invasive ductal carcinoma, ILC: invasive lobular carcinoma, TN: triple negative, CT: chemotherapy, ET: endocrine therapy, GI: gastrointestinal tracts, LN: contralateral lymph nodes

This study was carried out with approval from the ethics committee of Juntendo University Hospital (no.16-096) and all specimens were obtained after written informed consent had been obtained from the patients.

### Pathological diagnosis and immunohistochemistry

Pathological examinations were carried out at Juntendo University Hospital by two experienced pathologists. ER and PgR statuses were assessed semi-quantitatively and reported as positive when more than 1% of the nuclei of cancer cells showed staining. HER2 was judged to be positive if more than 10% of tumor cells showed strong staining of the entire cell membrane, or *HER2/neu* gene amplification was confirmed by fluorescence in situ hybridization.

Primary surgical specimens from the 61 patients were immunohistochemically investigated for CD44, CD24 and ALDH1 expressions and positive rates for these markers were semi-quantitatively assessed. A tumor was defined as CD44^+^/CD24^-^ when more than 5% of cancer cells showed a CD44-positive and CD24-negative staining pattern on the cell membrane with a double-staining method. ALDH1 was also defined as positive when more than 5% of cancer cells showed cytoplasmic staining. We employed the cut-off values established in previous studies [20, 21].

Since there were very few CD44^+^/24^+^ cells in our preliminary study using clinical samples, we also performed double-staining for CD44 and ALDH1 to reveal the staining patterns of these two markers.

Details of double staining; ALDH1/CD44: Antigen retrieval of paraffin-embedded tumor sections was performed in citrate buffer at 98C for 45 minutes. After removal of endogenous peroxidase activation by H_2_O_2_ with methanol, the sections were incubated with ALDH1 antibody (1:200 dilutions) overnight. Secondary staining utilized the anti-mouse secondary antibody (EnVision, Dako), followed by 3,3'-diaminobenzidine staining. Following re-antigen retrieval with TE buffer, samples were incubated with CD44 antibody (1:100 dilutions) overnight. After incubation with the secondary antibody, slides were stained with Vector^®^ SG Peroxidase Substrate (Vector Laboratories) for 15 minutes. CD44/24: The antigen retrieval methods were the same except that Tris-ethylene diamine tetra-acetic acid buffer was used for both staining procedures. CD24 antibody (1:50) was used for the first staining and CD44 (1:200) for the second.

For immunocytochemistry, 15-20x10^4^ sorted cells were seeded on a chamber slide and incubated for 48hrs. The cells were then fixed with 4% paraformaldehyde for 15 minutes before incubation with each antibody. Two hundred cancer cells were counted for each immunocytochemical assessment.

Details of antibodies; CD44: mouse monoclonal, clone 156-3C11 (Thermo Fisher Scientific), CD24: mouse monoclonal, clone SN3b (Thermo Fisher Scientific), ALDH1: mouse monoclonal, 44/ALDH (BD Biosciences).

### Statistical analysis

Using JMP V.10.0.1 (SAS Institute, Cary, NC, USA), Kaplan-Meier curves were drawn for patient outcomes and the log rank test was applied to compare the curves. We utilized the two-sided Student’s t test to examine unpaired data for comparison of CSC marker expressions between primary and metastatic tumors. A p value less than 0.05 was taken to indicate a statistically significant difference.

## Results

### Immunochemistry of CSCs extracted from a breast cancer cell line

To test whether the antibodies for IHC employed herein are feasible for evaluating cancer stemness, we first stained CSCs obtained from *in vitro* experiments (S1 Fig). CSCs were sorted from parental MDA-MB-231 cells, as described in our previous report [22], using flow cytometry with a combination of CD44 and CD24 antibodies, then split into chamber slides. The sorted cells had already been confirmed to have the ability to form xenografts, indicating the cells to be CSCs [22].

All CD44^+^/CD24^-^ cells reacted to the CD44 antibody but not to the CD24 antibody, while 88% and 22% of parental cells were positive for these markers, respectively. As to ALDH1, 8% of CD44^+^/CD24^-^ cells were positive for this protein, while 12% of parental cells expressed this marker.

We thus confirmed that the antibodies for IHC recognize CD44 and CD24 in *in vitro* cells and that the staining patterns of CSCs did indeed reflect CD44-positivity and CD24-negativity, despite these antibodies being different from those used for cell sorting.

### CD44^+^/24^-^ and ALDH1-positive rates differ according to intrinsic subtype

Next, primary tumors were immunohistochemically examined with double-staining for CD44 and CD24, or staining for ALDH1 alone. Representative images according to intrinsic subtype are shown in S2 Fig. The rates of being CD44^***+***^/24^-^ and ALDH1(+) were both highest in TN tumors (S3 Fig). Populations of CD44^***+***^/24^-^ cells were larger than those of ALDH1(+) cells, regardless of the intrinsic subtype. Among 38 luminal tumors, there were five HER2-positive cases and one of these five cases was CD44^***+***^/24^-^ and ALDH1(+). The rate was similar to that observed in HER2-negative luminal cases.

### CD44^+^/24^-^ tumors were associated with different patient outcomes according to ER status

**Fig 1:**
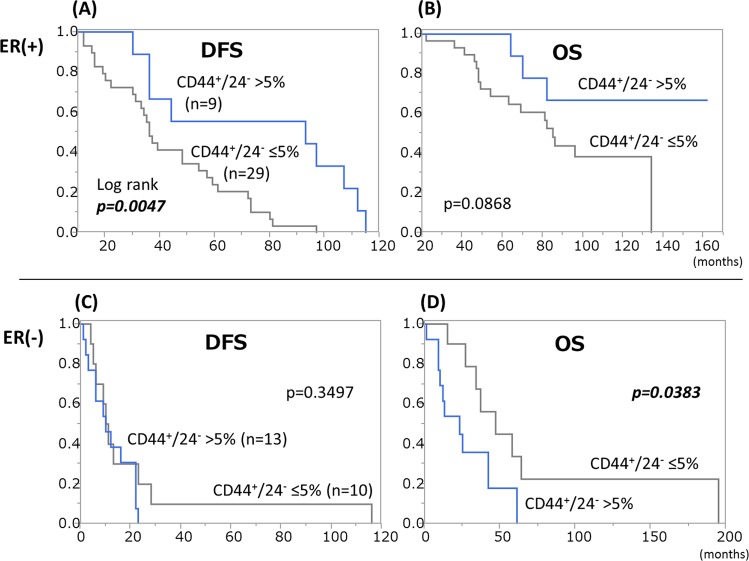
Population of CD44^+^/24^-^ cells and patient outcomes. CD44^+^/24^-^ tumors were associated with longer DFS (median 69 vs 36 months) in ER(+) patients (p<0.01). Conversely, OS was shorter in ER(-) patients (23 vs 47 months) (p<0.05).

ER(+) patients with CD44^+^/24^-^ tumors had significantly longer DFS than all other ER(+) patients during the 60-month median observation period (69 vs 36 months, p = 0.0047) (Fig 1). These results suggest that being CD44^+^/24^-^ may indicate a tumor with luminal-type characteristics, for which postoperative endocrine therapy might be effective.

On the other hand, CD44^+^/24^-^ tumors were associated with shorter OS in ER(-) patients (23 vs 47 months, p = 0.032). Chemotherapy was likely to be ineffective in these tumors, considering that the patients were given only chemotherapies for recurrences, although a variety of treatment regimens had been administered for recurrent breast cancer.

**Fig 2:**
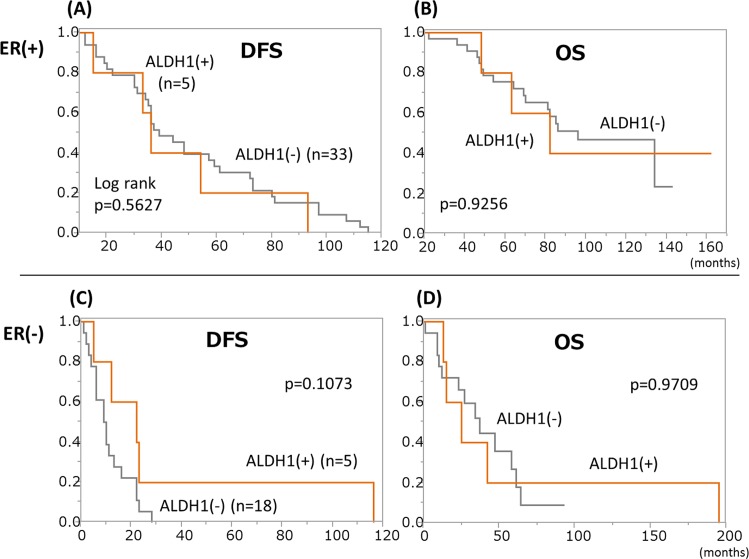
ALDH1 expression and patient outcomes. Among ER(-) cases, those with ALDH1(+) tumors tended to have longer DFS than those with ALDH1(-) tumors (median 23 vs 10 months), but the difference was not statistically significant.

As to ALDH1 staining, there was no survival difference between ALDH1(+) and (-) patients with ER(+) tumors (Fig 2). In ER(-) cases, ALDH1(+) tumors tended to be associated with longer DFS than ALDH1(-) tumors (23 vs 10 months), although the difference did not reach statistical significance. Taken together with the results shown in Figs 1D and 2C, our observations suggest that patients with CD44^+^/24^-^ and ALDH1(-) tumors have the poorest outcomes among those with ER(-) tumors. Indeed, median DFS and OS of patients with such tumors tended to be shorter than those of all other ER(-) patients (30 vs 65 months and 60 vs 116 months, respectively) in our dataset.

### CD44^+^/24^-^ tumors might be more resistant to chemotherapies in ER-negative patients

Interestingly, CD44^+^/24^-^ tumors showed different clinical behaviors according to ER status. We focused on the results of the ER(-) patients shown in Fig 1D and conducted further analyses. When OS after the development of distant metastasis was calculated, Kaplan-Meier curves revealed that CD44^+^/24^-^ patients had a much shorter OS than all of the other groups combined (median 7 vs 24 months, p = 0.006) (S4 Fig). Thus, we speculate that CD44^+^/24^-^ tumors might be more resistant to chemotherapies given after the development of distant metastasis.

### Staining patterns of CD44 and ALDH1 in individual tumors

**Fig 3:**
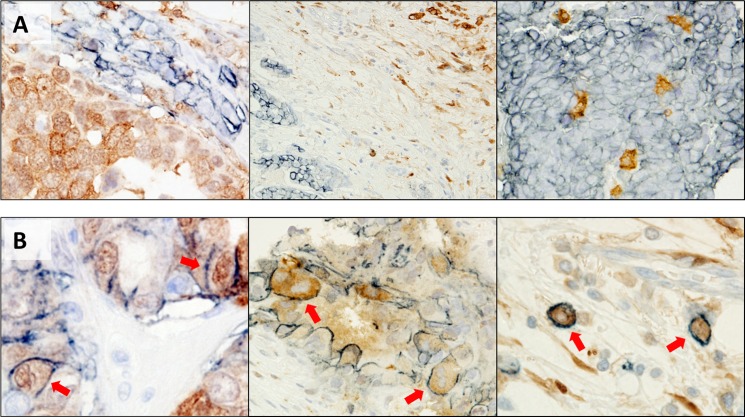
Staining patterns of CD44 and ALDH1 with a double-staining method. CD44 (cell surface) is stained blue and ALDH1 (cytoplasm) brown. (A) Typical staining patterns of these two markers are shown. (B) Rare double-positive cells are indicated by red arrows.

Finally, we employed IHC to examine CD44 and ALDH1 expressions in clinical samples, employing double-staining methods. We chose these two targets since the populations of CD44^+^/24^+^ cells were very small, no more than 5%, for examination by double-staining with CD44 and ALDH1. Expression patterns (areas showing staining) of CD44 and ALDH1 in individual tumors showed minimal overlap (Fig 3A). Even in a tumor containing numerous cells positive for either CD44 or ALDH1, or possibly both, there were very few individual double-stained cells, less than 1% (Fig 3B, arrows). Thus, we concluded that doubled-stained cells, i.e. those expressing both CD44 and ALDH1, were insufficient for further evaluation of their possible relationships with patient outcomes.

## Discussion

First, there appeared to be more double-stained CD44 and ALDH1 cells within a tumor than anticipated. However, expression patterns of these two proteins in a tumor were markedly different and there were very few double-stained cells, less than 1% of total cells, such that we could not evaluate relationships with patient outcomes. This discrepancy between the two protein expressions is consistent with previous reports [9], highlighting the need to be aware that different populations might be analyzed when only one of these markers is examined with IHC. Even in the large cohort study conducted by Ricard et al., CD44^+^/24^-^ cells and ALDH1-positive cells were separately evaluated [17]. Although triple-staining of all three markers (CD44, CD24 and ALDH1) should be conducted for precise evaluation, we instead employed double-staining of CD44 and ALDH1 and revealed populations of cells expressing these two proteins to show virtually no overlap.

The clinical importance of positivity for CSC markers, evaluated with IHC, remains uncertain. Considering their plasticity, whether the cells positive for these markers are actually CSCs is unknown. Even when positive cells identified employing the same antibodies are extracted from clinical samples for *in vitro* and *in vivo* cultures, the results cannot be directly compared with data obtained using cell lines. Even if IHC evaluation precisely reflects cancer stemness, the overall interpretation of such data would still be challenging. However, the patients showing increased CSC markers did not always have poorer outcomes, according to another study [23]. Meanwhile, CD44^+^/24^-^ cells were enriched in only 5 of 13 major established cell lines [7], despite most of these cells having been extracted from metastatic sites. Considering the plasticity of CSCs, the presence of an abundance of CSC marker-positive cells does not appear to reflect more malignant tumor histology. Thus, we still do not fully understand the importance of high rates of these markers within a tumor.

In our present study, the combination of CD44 and CD24 was superior to ALDH1 alone in terms of reflecting patient outcomes and treatment effects. CD44^+^/24^-^ tumors were associated with longer DFS in ER(+) patients, suggesting that such tumors might be more responsive to endocrine therapy than other ER(+) tumors. On the other hand, in ER(-) patients, CD44^+^/24^-^ tumors were associated with shorter OS (Fig 1D) and the difference became more obvious when Kaplan-Meier curves were drawn for OS after the development of distant metastasis (S3 Fig). A previous study by Ricard et al. also demonstrated that patients with primary tumors containing abundant CD44^+^/24^-^ cells had significantly poorer outcomes but this trend was observed only in patients with TN tumors [17]. Our results indicate that patients with such tumors should be given chemotherapies starting with the strongest drug regimen available. Moreover, this population might consist of good candidates for clinical trials using new drugs, such as treatments targeting CSCs themselves [24, 25].

Based on the results obtained in this study, the CD44^+^/24^-^ and ALDH1(-) pattern might be a prognostic marker suggesting poor outcomes for ER(-) patients. However, the number of patients in this study was insufficient for drawing firm conclusions. Further studies, with more subjects, are needed to test the usefulness of combining these markers to predict patient outcomes.

## Conclusions

Staining patterns of CD44(+) and ALDH1(+) cells within tumors were markedly different. Also, populations of CD44^+^/24^-^ and ALDH1(+) cells differed according to ER status. Thus, we must be cautious when interpreting IHC results using CSC markers. Our results raise the possibility of CD44^+^/24^-^ being a good prognostic marker, one which would allow treatment effects and outcomes to be predicted in patients with recurrent breast cancer.

## Supporting Information

S1 Fig Immunocytochemistry of CSCs. CSCs from parental MDA-MB-231 cells were stained with CD44, CD24 and ALDH1 antibodies on chamber slides. Results of CD44^+^/24^-^ cells are presented. Also, control images for both proteins are in the bottom half of the figure. Weak cytoplasmic staining was observed in some of the negative control cells, probably due to: only background staining; small amounts of these proteins possibly existing in the cytoplasm; differences in recognitions of the antibodies. In summary, we confirmed that the antibodies employed for IHC identified the same surface proteins as those used for cell sorting.
(PDF)

S2 Fig Representative images of Hematoxylin-Eosin, CD44/24 and ALDH1 staining according to intrinsic subtype. There are no apparent differences in histological structures or staining patterns among subtypes.
(PDF)

S3 Fig Rates of CD44^+^/24^-^ and ALDH1(+) tumors according to intrinsic subtype. Among primary tumors, the rates of CD44^***+***^/24^-^ and ALDH1(+) were both highest in TN tumors. Populations of CD44^***+***^/24^-^ cells were larger than those of ALDH1(+) cells, regardless of intrinsic subtype. Among luminal tumors, five HER2-positive luminal cases showed trends similar to those observed in HER2-negative luminal cases.
(PDF)

S4 Fig OS in ER(-) patients after the development of distant metastasis. CD44^+^/24^-^ tumors were associated with much shorter OS in ER(-) patients after the development of distant metastasis.
(PDF)

S1 Table Clinicopathological features and IHC results of all 61 patients. (XLSX)
