# Supplementary figures and images for: Combination of Cancer Stem Cell Markers CD44 and CD24 Is Superior to ALDH1 as a Prognostic Indicator in Breast Cancer Patients with Distant Metastases
Source: PLoS One. 2016 Oct 21;11(10):e0165253. doi: 10.1371/journal.pone.0165253 (PMC5074575; doi:10.1371/journal.pone.0165253)

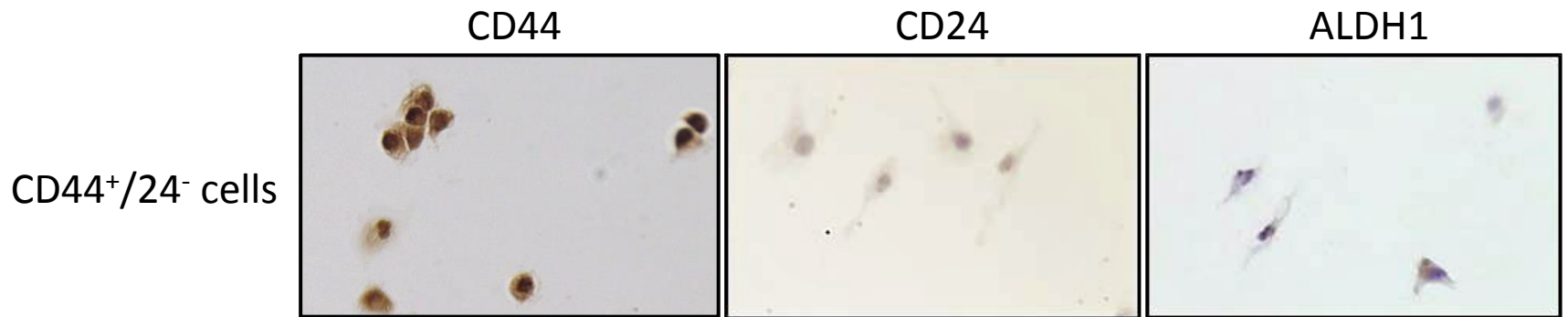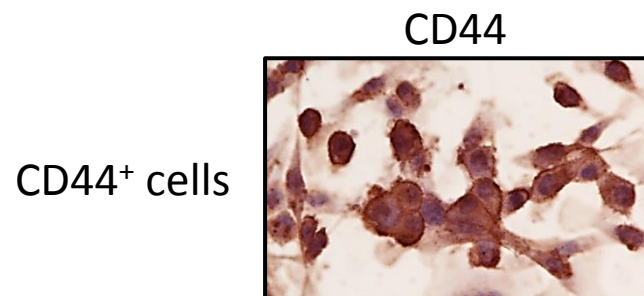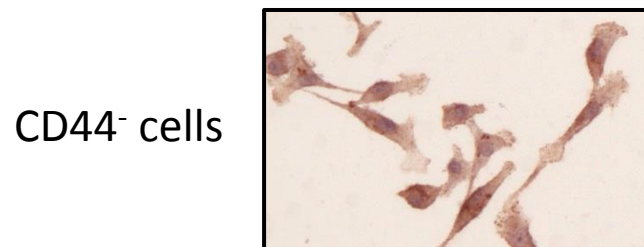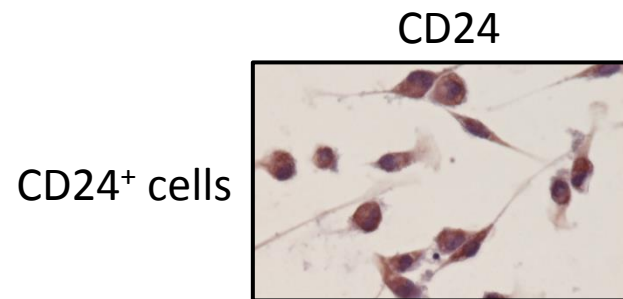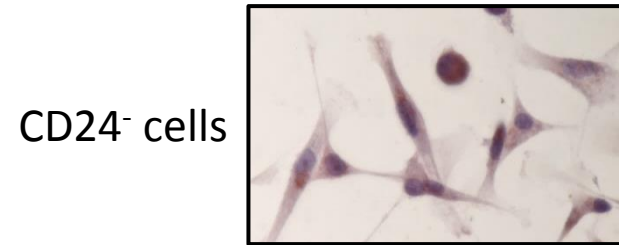

Supplement: S1 Fig — CSCs from parental MDA-MB-231 cells were stained with CD44, CD24 and ALDH1 antibodies on chamber slides. Results of CD44+/24- cells are presented. Also, control images for both proteins are in the bottom half of the figure. Weak cytoplasmic staining was observed in some of the negative control cells, probably due to: only background staining; small amounts of these proteins possibly existing in the cytoplasm; differences in recognitions of the antibodies. In summary, we confirmed that the antibodies employed for IHC identified the same surface proteins as those used for cell sorting. (PDF) [file pone.0165253.s001.pdf]

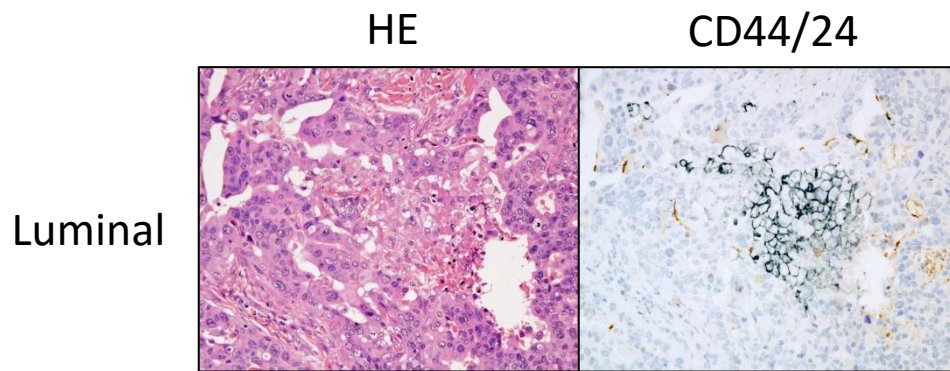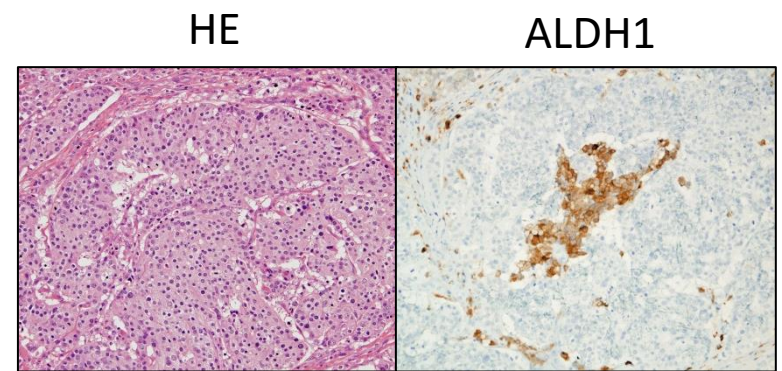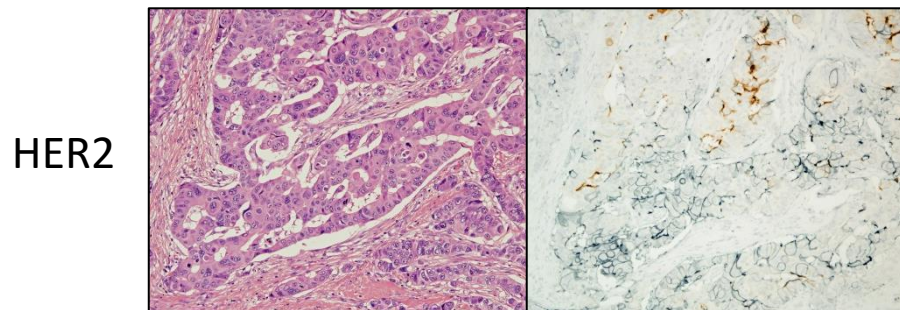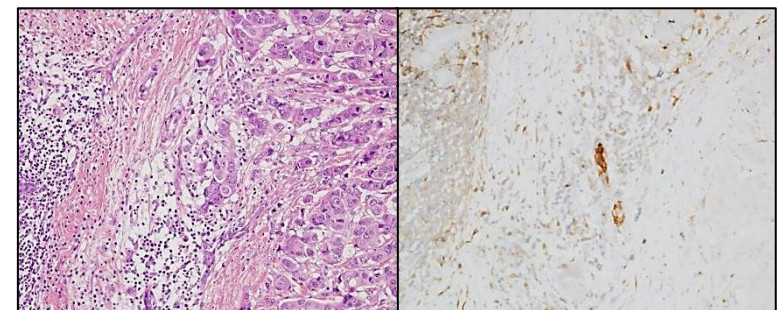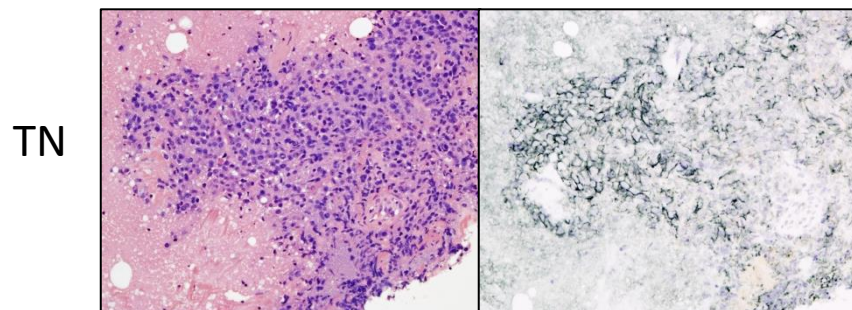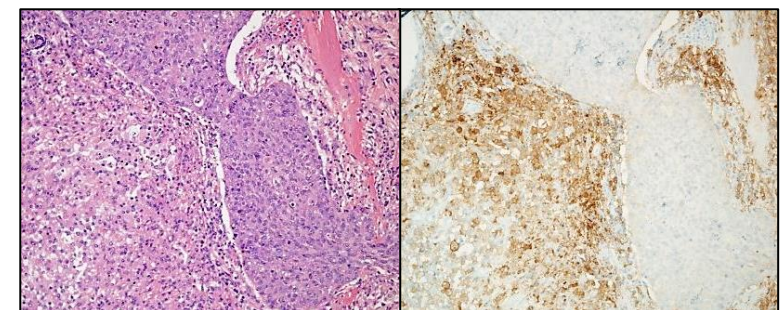

Supplement: S2 Fig — There are no apparent differences in histological structures or staining patterns among subtypes. (PDF) [file pone.0165253.s002.pdf]

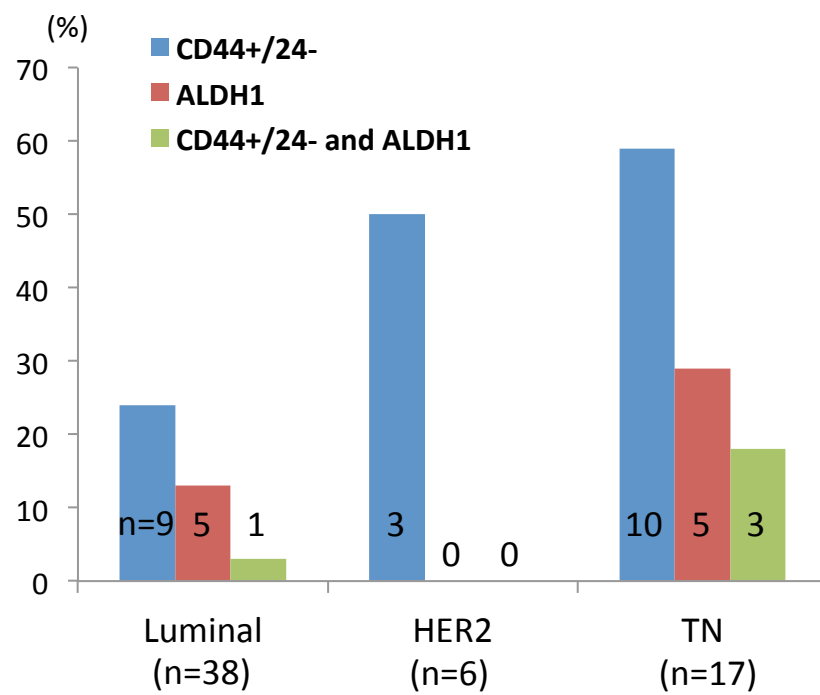

Supplement: S3 Fig — Among primary tumors, the rates of CD44+/24- and ALDH1(+) were both highest in TN tumors. Populations of CD44+/24- cells were larger than those of ALDH1(+) cells, regardless of intrinsic subtype. Among luminal tumors, five HER2-positive luminal cases showed trends similar to those observed in HER2-negative luminal cases. (PDF) [file pone.0165253.s003.pdf]

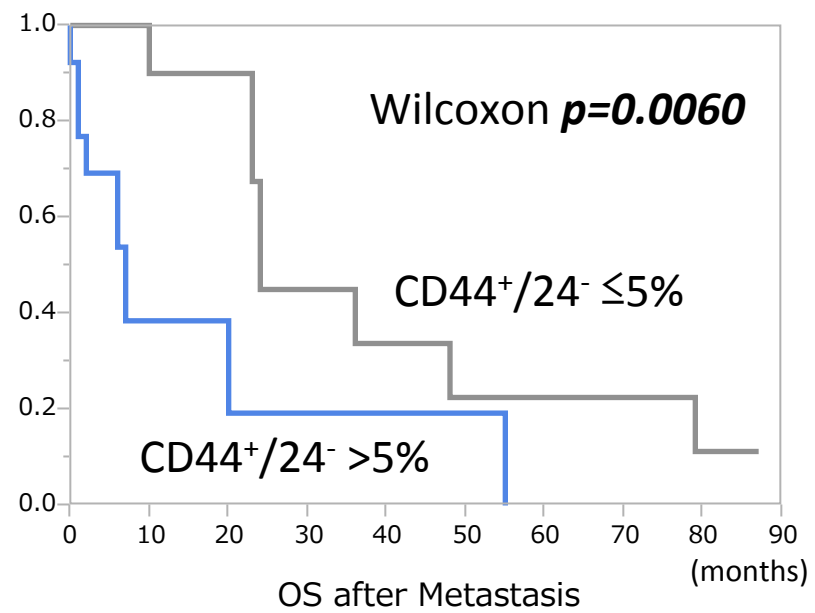

Supplement: S4 Fig — CD44+/24- tumors were associated with much shorter OS in ER(-) patients after the development of distant metastasis. (PDF) [file pone.0165253.s004.pdf]
